# A Case of Ectopic Vas Deferens With Contralateral Vesicoureteral Reflux Causing Recurrent Pyelonephritis and Epididymitis

**DOI:** 10.7759/cureus.77209

**Published:** 2025-01-09

**Authors:** Kenichi Kobayashi, Kazuyoshi Johnin, Akinori Wada, Kazuaki Yamanaka, Susumu Kageyama

**Affiliations:** 1 Department of Urology, Shiga University of Medical Science, Otsu, JPN

**Keywords:** congenital anomalies of the kidney and urinary tract (cakut), ectopic vas deferens, infertility, mesonephric duct abnormality, recurrent epididymitis, vasectomy, vesicoureteric reflux (vur), wolffian duct anomalies

## Abstract

Ectopic vas deferens is a rare congenital anomaly and can be associated with urinary tract and genital anomalies. Therefore, patients can present with a variety of symptoms. We present the case of a four-year-old uncircumcised male patient with ectopic vas deferens and contralateral vesicoureteral reflux (VUR). Our patient had been caused breakthrough pyelonephritis and recurrent epididymitis alternately, and this made it difficult for us to reach an accurate diagnosis. The left vas deferens was connected to the bladder, and it was opening next to the left ureteral orifice. A bilateral ureteroneocystostomy and left vasectomy were performed, and the patient has been doing well and has had no further urinary tract infections (UTIs) or epididymitis.

## Introduction

Ectopic vas deferens is a rare congenital anomaly with less than 70 cases reported [1]. The patients can present with varying symptoms that depend on not only the location of ectopic vas insertion but are also potentially associated with urinary tract and genital anomalies [2]. Therefore, it can make accurate diagnosis of pathological conditions difficult in clinical practice. We present a case of an ectopic vas deferens and contralateral vesicoureteral reflux (VUR). These conditions uniquely caused breakthrough pyelonephritis and recurrent epididymitis alternately.

## Case presentation

**Figure 1:**
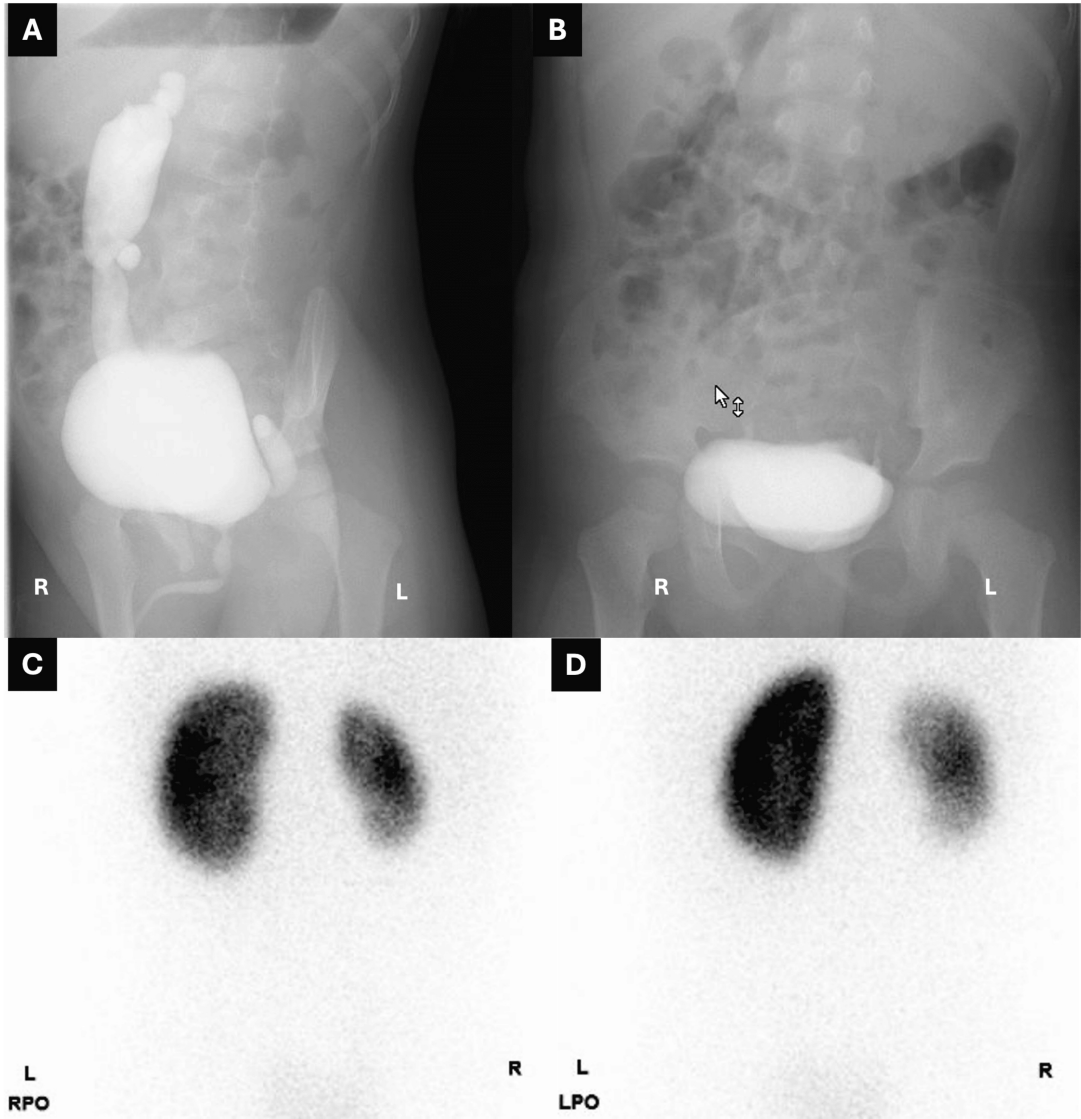
Voiding cystourethrography (VCUG) and renal scintigraphy images The VCUG disclosed grade 4 right and grade 1 left VUR. However, we should have noticed that the left side seems to be atypical, as grade 1 VUR retrospectively, bilateral ureteroneocystostomy had been scheduled. A: Voiding phase; B: Post-voiding image of frontal view 99mTc-dimercaptosuccinic acid (DMSA) static renal scintigraphy showed a right atrophic kidney and scarring. C: Right posterior oblique (RPO); D: Left posterior oblique (LPO)

A four-year-old boy was referred for recurrent urinary tract infection (UTI). He had not undergone circumcision but was able to retract the foreskin. Both testes were descended. He had achieved potty training and had no constipation. The spinal cord workup had been performed because of a sacral dimple. The MRI showed no significant findings in his spinal cord and lower abdomen. Voiding cystourethrography (VCUG) disclosed grade 4 right and grade 1 left VUR (Figures 1A, 1B). 99mTc-dimercaptosuccinic acid (DMSA) static renal scintigraphy showed severe renal atrophy and scarring on the right kidney (Figures 1C, 1D). 

**Figure 2:**
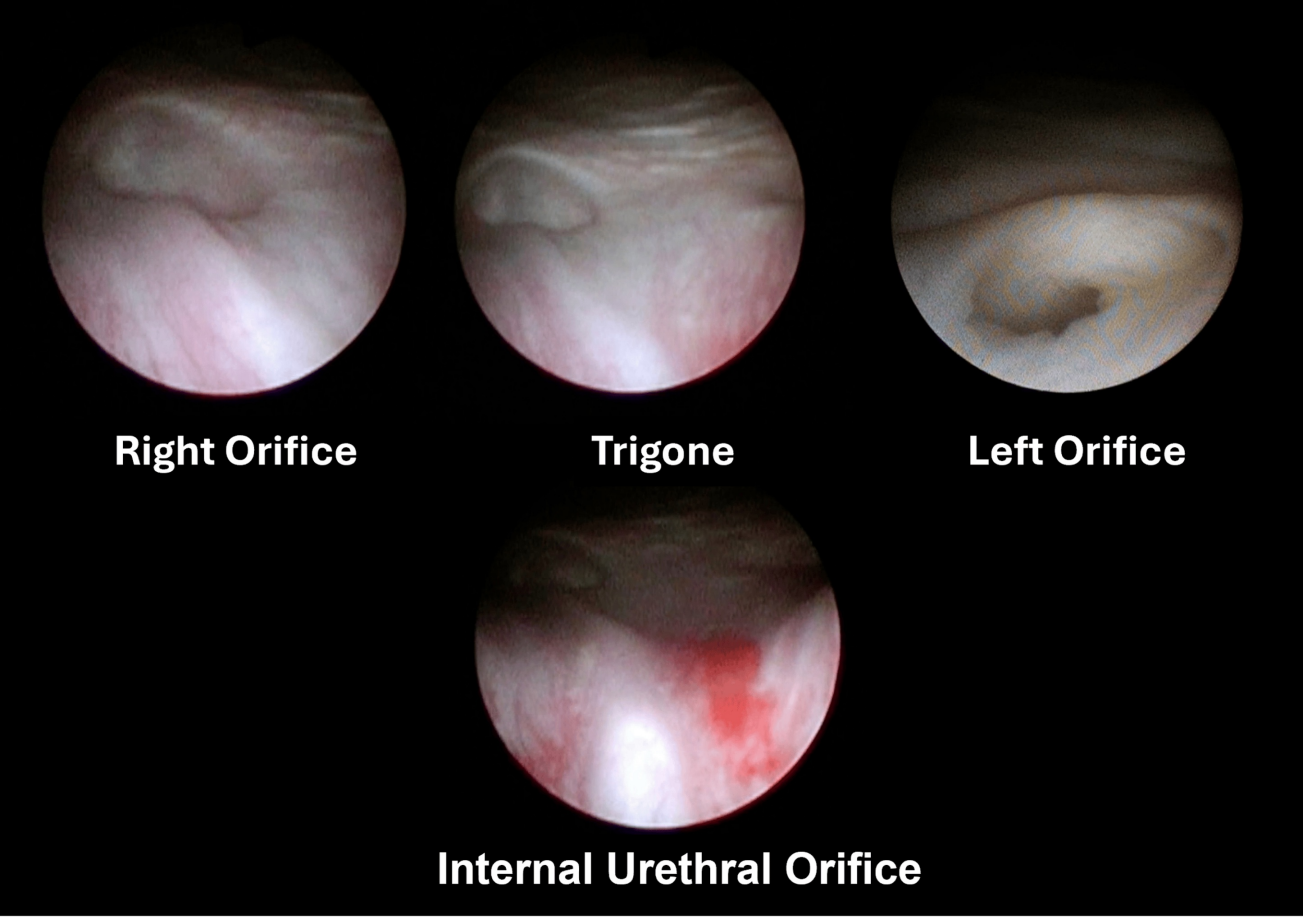
Cystourethroscopy images Cysturethrscopy was performed since concomitant urethral stricture had been suspected. Bladder trigone atrophy, hypoplasia of the posterior urethra, and no urethral stricture were observed.

**Figure 3:**
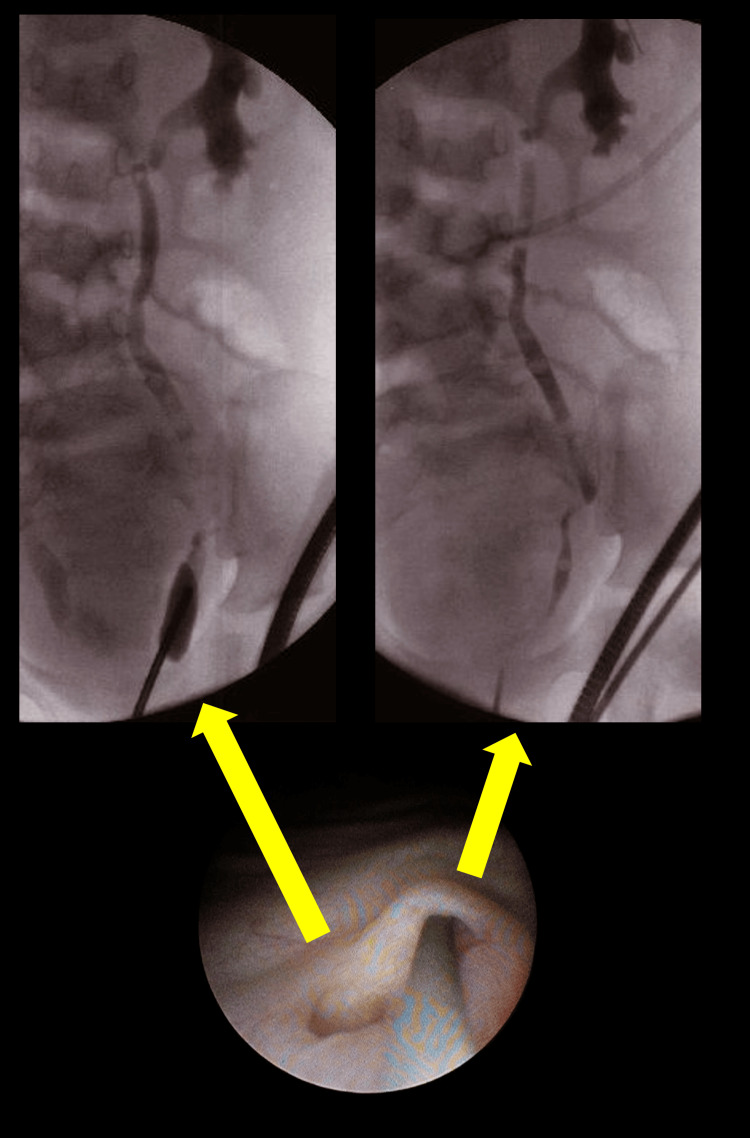
Retrograde urography images Two orifices were identified on the left side. The upper side was identified as the left ureteral orifice by retrograde urography. The lower side, diagnosed as a grade 1 vesicoureteral reflux, remained unknown. We speculated that the left ectopic vas deferens opened into the bladder, causing recurrent epididymitis.

We had scheduled a bilateral ureteroneocystostomy based on the diagnosis of bilateral VUR. However, he experienced repeated epididymitis before surgery. Cystoscopy and retrograde urography were performed under general anesthesia since concomitant urethral stricture had been suspected. No urethral stricture was observed, and it revealed bladder trigone atrophy and two side-by-side orifices (Figures 2, 3).

One was identified as the normal ureteral orifice, and the other had an enlarged short duct, which was considered grade 1 VUR with no connection to the ureter. We speculated that the left ectopic vas deferens opened into the bladder, causing recurrent epididymitis. We performed open surgery to correct these anomalies.

**Figure 4:**
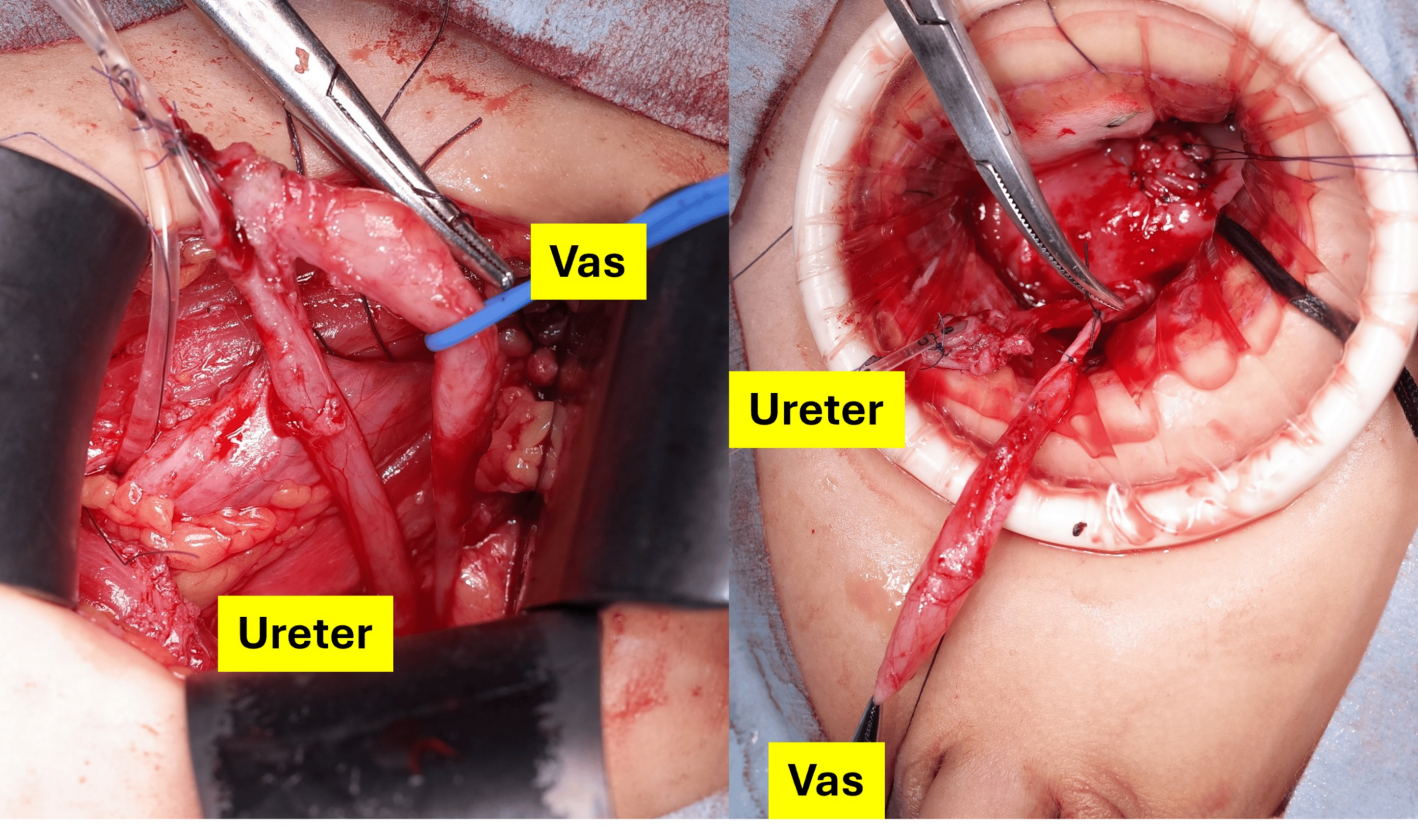
Intraoperative findings The left ureter and vas deferens (vas) were identified.

Transvesical excision allowed dissection of the duplex orifices from the bladder wall. We dissected the double duct from the left extravesical wall. One duct was confirmed as the left ureter, and the other was a vas deferens connected to scrotal structures (Figure 4).

The vas deferens was excised from the ureter, the stump was ligated, and the left ureter was pulled back into the bladder from the original hiatus. The right ureter was then fully mobilized, and both hiatuses were plicated to the appropriate size. New submucosal tunnels were created trans-trigonally on the opposite side of each other. Ureters were transposed through the new submucosal tunnel and reimplanted into the bladder wall. The bladder was closed in three layers, and a Foley catheter was inserted and remained in place for two days. The patient was discharged on the third day after surgery.

The patient has remained healthy since surgery with no further pyelonephritis and epididymitis.

## Discussion

We present a rare congenital anomaly of an ectopic vas deferens associated with contralateral VUR, which led to recurrent pyelonephritis and epididymitis in a young patient.

Ectopic vas deferens is a rare congenital anomaly with about 70 cases described in the literature [1]. The most common location for ectopic vas insertion is the ipsilateral ureter [3-5], and inserting into the bladder is less common. Limited evidence shows the high frequency of coincidence of other urogenital anomalies and anorectal anomalies [6-8]. Matsumoto et al. showed that 15 of 25 cases of ectopic vas deferens have been associated with anorectal abnormalities [3]. Concomitant hypospadias and dysplastic kidney are also well-documented urogenital anomalies. 

Diagnosing ectopic vas deferens can be challenging due to its rarity and the variability in clinical presentation. The patients can present with varying symptoms which are affected by the location for ectopic vas insertion and associated diseases. Some patients experienced severe bacterial infection while some have not been found since examination for infertility. 

Magnetic resonance imaging can be a valuable imaging modality to evaluate anatomical abnormalities in the genitourinary tract and should be performed in a patient considered for surgical intervention. However, it is rare to detect ectopic vas by MRI, especially in pediatric patients; only a few adult case reports have been documented on MRI with findings [5-8].

**Figure 5:**
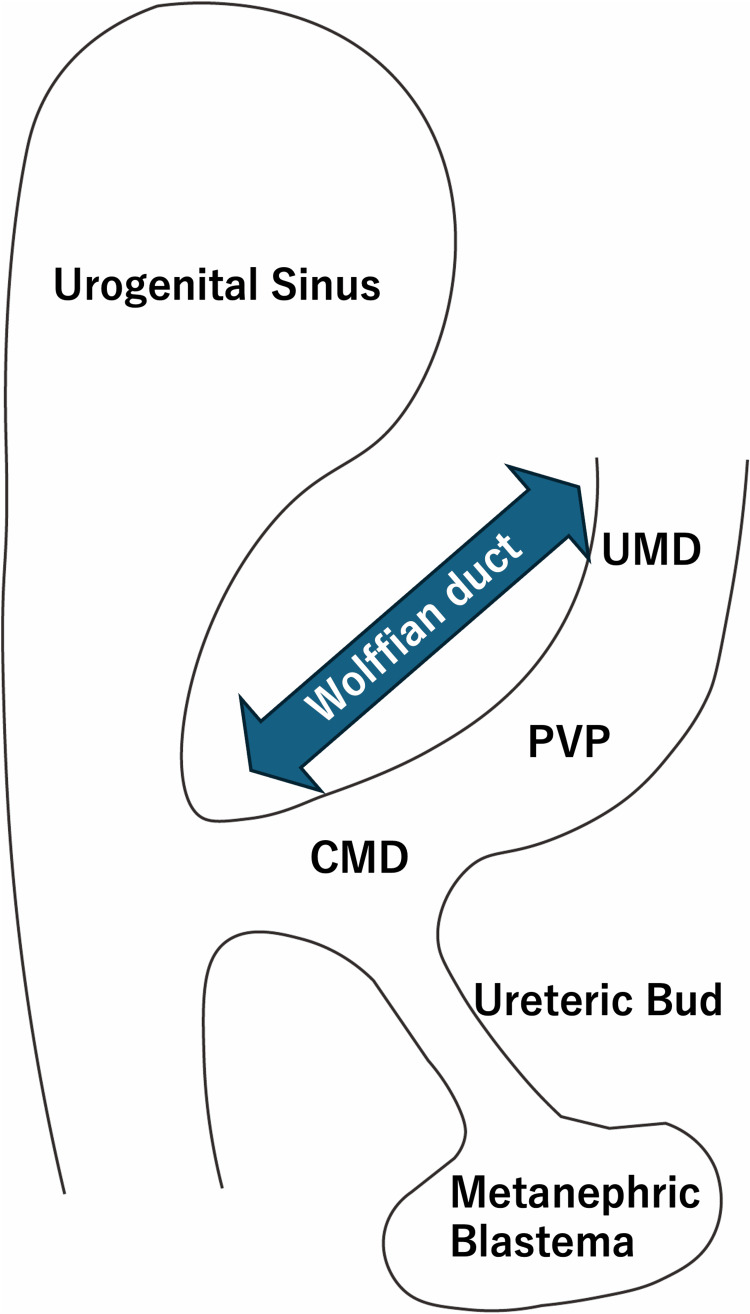
Schema of the Wolffian duct CMD: common mesonephric duct; PVP: proximal vas precursor; UMD: upper mesonephric duct Figure 5 has been created by the authors.

The Wolffian duct serves as the precursor for both the ureter and the vas deferens [6]. The ureteric bud arises from the distal Wolffian duct, which is called the common mesonephric duct (CMD). Proximal vas precursor (PVP) was just proximal to the CMD, and the precursor to the distal vas and epididymis is the upper mesonephric duct (UMD), located proximal to the PVP on the Wolffian duct (Figure 5).

It is said that the PVP encroaches onto the upper budding segment of the CMD, and then the PVP follows the ureteral bud, resulting in an ectopic vas deferens inserted into the bladder.

Bladder trigone hypoplasia is inevitable because the ureteric bud and PVP have no distance in those cases. It can be strongly associated with the occurrence of VUR and voiding dysfunction. The co-existence of contralateral VUR complicates clinical practice, although they have a reasonably potential shared embryologic origin or a coincidental but complex interaction of genitourinary anomalies. Vesicoureteral reflux, particularly of high grade, poses a risk of renal damage and recurrent UTI, as observed in this patient. The anatomical anomalies disrupted the normal urinary flow, leading to both pyelonephritis and epididymitis. While VUR is common in pediatric populations, the occurrence of ectopic vas deferens is less well-documented. Therefore, it might be difficult to notice the signs of ectopic vas deferens while we always take care of VUR in children with febrile UTIs. In our case, we had misdiagnosed it as bilateral VUR, although the duct of the left side in urography had seemed to be atypical as grade 1 VUR retrospectively.

Another key point in the pathophysiology of ectopic vas deferens is the relationship with seminal vesicle agenesis [9]. Since the seminal vesicles arise from the PVP, their absence or abnormality is a consistent feature with ectopic vas deferens. This has significant implications for future reproductive potential, as the lack of seminal vesicles impairs ejaculation, essentially rendering the patient infertile. In this case, the patient would likely include sterility [10], and vasectomy can be a reasonable option for preventing recurrent epididymitis.

## Conclusions

We experienced a rare case of ectopic vas deferens with contralateral VUR causing recurrent pyelonephritis and epididymitis. Ectopic vas deferens can be associated with other urogenital anomalies and anorectal anomalies, and concomitant VUR made it difficult to diagnose it in our case.　

It is difficult to preserve ejaculation ability in patients with ectopic vas deferens. Therefore, vasectomy can be a safe and effective treatment option for preventing repeat epididymitis with an ectopic vas deferens.
